# Biodistribution of neutralizing monoclonal antibodies IgG1 b12 and LALA in mucosal and lymphatic tissues of rhesus macaques

**DOI:** 10.1186/1742-4690-9-S2-P204

**Published:** 2012-09-13

**Authors:** AJ Hessell, E Epson, B Moldt, E Rakasz, S Pandey, WF Sutton, Z Brower, VM Hirsch, DR Burton, NL Haigwood

**Affiliations:** 1 Oregon Health & Science University, Oregon National Primate Res Ctr, Beaverton, OR, USA; 2 The Scripps Research Institute, La Jolla, CA, USA; 3 University of Wisconsin-Madison, Madison, WI, USA; 4 NIAID, NIH, Bethesda, MD, USA; 5 The Scripps Research Institute and IAVI Neutralizing Antibody Center, La Jolla, CA, USA

## Background

HIV-1 transmission occurs predominantly through mucosal surfaces. In macaque models of SHIV mucosal transmission, passive administration of the monoclonal neutralizing antibody IgG1 b12 (b12) can protect against virus infection. However, an Fc variant of b12, deficient in FcγR and complement binding, (LALA) had diminished protective capacity. Concentrations and kinetics of b12 and LALA in serum and in mucosal secretions were determined in the protection studies, but the biodistribution and kinetics of the antibodies in mucosal and lymphatic tissues and around the site of viral challenge have not been assessed.

## Methods

We conducted a pilot study to develop protocols to process macaque tissue specimens and to detect and quantify passively transferred neutralizing antibodies (NAbs) in tissue homogenates. To confirm that transport of LALA to the site of challenge was not altered from that of b12, we obtained secretions and tissue specimens from rhesus macaques that had been passively infused with either b12 or LALA at 24 hours prior to necropsy, matching the time of challenge in the protection study.

## Results

We quantified passively administered b12 and LALA in a variety of macaque mucosal and lymphoid tissues and assessed the neutralization capacity of the NAbs localized in vaginal and rectal sites typically exposed to virus in challenge studies. We demonstrated that the rapid distribution and broad delivery to lymphoid and mucosal tissues of both b12 and LALA are equivalent.

## Conclusion

We developed and utilized a methodology to evaluate the transudation of transferred antibody into mucosal and lymphatic tissues and showed that the reduction in the protective capacity of LALA compared to b12 cannot be attributed to a differential effect of biodistribution. This methodology will be useful to describe the pharmacokinetics of newly discovered MAbs and may inform about the characteristics of therapeutic antibodies or antibodies to elicit by vaccination.

